# Dual Inhibition of FAAH and MAGL Counteracts Migraine-like Pain and Behavior in an Animal Model of Migraine

**DOI:** 10.3390/cells10102543

**Published:** 2021-09-26

**Authors:** Rosaria Greco, Chiara Demartini, Miriam Francavilla, Anna Maria Zanaboni, Cristina Tassorelli

**Affiliations:** 1 Unit of Translational Neurovascular Research, IRCCS Mondino Foundation, via Mondino 2, 27100 Pavia, Italy; chiara.demartini@mondino.it (C.D.); miriam.francavilla@mondino.it (M.F.); annamaria.zanaboni@mondino.it (A.M.Z.); cristina.tassorelli@mondino.it (C.T.); 2 Department of Brain and Behavioral Sciences, University of Pavia, via Bassi 21, 27100 Pavia, Italy

**Keywords:** hyperalgesia, FAAH, MAGL, migraine

## Abstract

The endocannabinoid system exerts an important role in pain processing and modulation. Modulation of the system with hydrolase inhibitors of anandamide (AEA) or 2-arachidonyl glycerol (2-AG) has proved effective in reducing migraine-like features in animal models of migraine. Here, we investigated the effect of dual inhibition of the AEA and 2-AG catabolic pathways in the nitroglycerin-based animal model of migraine. The dual inhibitor JZL195 was administered to rats 2 h after nitroglycerin or vehicle injection. Rats were then exposed to the open field test and the orofacial formalin test. At the end of the tests, they were sacrificed to evaluate calcitonin gene-related peptide (CGRP) serum levels and gene expression of CGRP and cytokines in the cervical spinal cord and the trigeminal ganglion. The dual inhibitor significantly reduced the nitroglycerin-induced trigeminal hyperalgesia and pain-associated behavior, possibly via cannabinoid 1 receptors-mediated action, but it did not change the hypomotility and the anxiety behaviors induced by nitroglycerin. The decreased hyperalgesia was associated with a reduction in CGRP and cytokine gene expression levels in central and peripheral structures and reduced CGRP serum levels. These data suggest an antinociceptive synergy of the endocannabinoid action in peripheral and central sites, confirming that this system participates in reduction of cephalic pain signals.

## 1. Introduction

The endocannabinoid system exerts many physiological functions among which resides the important role in pain processing and modulation [1]. This endogenous system comprises the cannabinoid (CB) receptors, their ligands, and the enzymes designated to the synthesis and catabolism of endocannabinoids. Endocannabinoids induce anti-nociception when exogenously administered. This has been demonstrated for the two best characterized endocannabinoids, anandamide (AEA) and 2-arachidonyl glycerol (2-AG). The endocannabinoids act as retrograde or local neurotransmitters by signal transduction of the CB receptors. CB receptors are 7-transmembrane G-protein-coupled receptors (GPCRs) and play a key role in peripheral, spinal, and supraspinal nociception, including ascendant and descendent pain pathways [2]. CB1 are primary expressed in neurons, at the presynaptic level, throughout the brain and spinal cord; whereas CB2 are primarily found in immune cells, although they are located in the nervous system as well [1]. AEA showed anti-nociceptive actions in experimental models of acute and chronic pain [3]. In the same way, 2-AG decreased pain behavior in the tail-flick and formalin test [4,5]. AEA and 2-AG are indeed present in key central and peripheral areas involved in the modulation and integration of nociceptive inputs, including the skin, dorsal root ganglia, spinal cord, periaqueductal gray (PAG), and rostral ventromedial medulla. Moreover, local administration of 2-AG or AEA causes anti-nociceptive effects in an inflammatory pain model induced by intra-plantar formalin injection, confirming the peripheral action of endocannabinoids by CB1 receptors [5]. The restricted increase of AEA and 2-AG levels by specific inhibitors of the catabolic enzymes is proposed to produce localized action, thus limiting adverse side effects compared with the exogenous cannabinoids [6,7,8]. Interestingly, dual inhibition of fatty acid amide hydrolase (FAAH) and monoacylglycerol lipase (MAGL) has also shown analgesic effects in different animal models of pain [9,10]. Specifically, preclinical studies reported that the inhibition of these enzymes by specific FAAH or MAGL inhibitors induces anti-nociceptive effects with fewer or no side effects, compared to exogenous cannabinoids, in different animal models of inflammatory and neuropathic pain [1,9,10,11,12]. The compound JZL195, for instance, has been described as a strong inhibitor of both FAAH and MAGL [7] that increases AEA and 2-AG levels, respectively, in specific brain areas, but also those of the endocannabinoid-like congeners, palmitoylethanolamide (PEA) and oleoylethanolamide (OEA), causing antinociception and analgesia in diverse pain models [7,13,14]. Additionally, JZL195 may be more effective in alleviating neuropathic pain compared with selective FAAH or MAGL inhibitors or cannabinoid receptor agonists [13]. 

Migraine is a neurovascular disorder affecting about 12% of the general population, and the biological origin of migraine is multifactorial with a complex pathophysiology. One of the main mechanisms underlying migraine pain is the activation and sensitization of the trigeminovascular system, comprising trigeminal and autonomous innervation of meninges, local blood vessels, and mast cells [15,16]. The activation of this pathway is accompanied by abnormal nociceptive processing, central sensitization, and changes in cerebrovascular and immune functions [17]. 

Clinical and preclinical evidence suggests a link between dysregulation of endocannabinoid signaling and migraine headache and migraine patients showing reduced endocannabinoid activity [18,19,20,21,22,23,24,25]. Previous studies have demonstrated that modulation of the endocannabinoid system by selective inhibitors of the endocannabinoids’ catabolic enzymes—FAAH and MAGL—may represent a new therapeutic strategy for migraine [26,27,28,29,30]. In particular, peripheral FAAH inhibition by URB937 increases the levels of AEA in trigeminal ganglia and reduces the trigeminal hyperalgesia induced by nitroglycerin (NTG) administration, used as an animal model of migraine [30]. The modulation of trigeminal hyperalgesia was associated with a reduced activation of the neuropeptidergic (i.e., calcitonin gene-related peptide, CGRP) and inflammatory (e.g., interleukin-6) pathways triggered by NTG. Moreover, MAGL inhibition proved more effective in preventing trigeminal hyperalgesia induced by NTG administration [28]. Taken together, our studies suggest a therapeutic potential of both endocannabinoids by dual FAAH/MAGL inhibition in migraine pain.

Here, we tested the dual FAAH/MAGL inhibitor JZL195 in an animal model specific for migraine as a pharmacological probe to evaluate the impact of simultaneous inhibition of AEA and 2-AG degradation on the trigeminal system. More specifically, we evaluated the effect of JZL195, an FAAH/MAGL inhibitor, on (i) NTG-induced trigeminal hyperalgesia at the orofacial formalin test; (ii) anxiety-like behavior and spontaneous locomotor activity; and (iii) molecular mediators of the potential JZL195 anti-migraine effect.

The data presented in this study suggest that the dual inhibition of AEA and 2-AG catabolism reduces NTG-induced trigeminal hyperalgesia, representing a new therapeutic target for the treatment of migraine. 

## 2. Materials and Methods

### 2.1. Animals

Male Sprague–Dawley rats (200–270 g, Charles River Laboratories, Italy) were housed in the animal facility of University of Pavia in groups of two per cages under controlled conditions (i.e., temperature 21–22 °C, 60–50% relative humidity) and 12/12 h light cycle (with lights on at 7.00 a.m.). Food and water were available ad libitum. All procedures were approved by the Italian Ministry of Health (376/2020- PR) and performed in agreement with the guidelines of the European Community Directive 2010/63/EU of 22 September 2010. Upon arrival, animals were habituated to the housing conditions for 1 week before the experimental testing.

### 2.2. Drugs and Treatment

**Figure 1:**
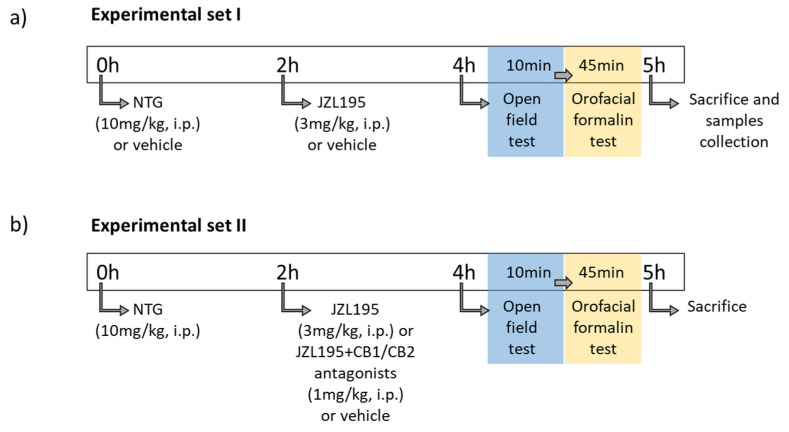
Experimental timeline for the treatment and testing procedures. (**a**) In the first experimental set, JZL195 (or its vehicle) was administered 2 h after NTG (or its vehicle) and 2 h before the open field test (of 10 min duration); immediately after the open field, rats were injected with formalin (50 μL, s.c.) and the orofacial formalin test was performed (45 min duration). At the end of the test, rats were sacrificed by decapitation and samples collected for ex vivo analysis. (**b**) The second experimental set was achieved to evaluate CB1 and CB2 involvement: JZL195 was administered in combination with AM251 (CB1 antagonist, 1 mg/kg, i.p.) or AM630 (CB2 antagonist, 1 mg/kg, i.p.) 2 h after NTG and 2 h before the behavioral tests.

NTG (Bioindustria L.I.M., Novi Ligure, Italy) was prepared from a stock solution of 5.0 mg/1.5 mL dissolved in 27% alcohol and 73% propylene glycol. The NTG solution was diluted in saline (0.9% NaCl) to reach a final concentration of alcohol of about 6% and propylene glycol of 16% and administered intraperitoneally (i.p.) at a dose of 10 mg/kg. The NTG vehicle contained saline, alcohol 6%, and propylene glycol 16% [30]. JZL195, an inhibitor of both FAAH and MAGL [7], was purchased from Cayman Chemical Company (Ann Arbor, MI), dissolved in Tween-80/polyethylene glycol 200/saline (10/10/80), and administered intraperitoneally at a dose of 3 mg/kg dissolved in a volume of 2 mL/kg, 2 h before testing (Figure 1), in accordance with previous studies [31,32,33].

The 3 mg/kg dose of JZL195 was chosen based on available data on its anti-nociceptive effects in animal models of inflammatory and neuropathic pain [9,13]. As regards the choice of administering JZL 195 2 h before the behavioral testing, this timing represented the best trade-off, when considering that the 3 mg/kg dose of JZL 195 induces an antinociceptive effect 1 and 2 h after i.p. injection [9,13] and the increase in AEA and 2-AG brain levels occurs from 30 min to 4 h after the injection [7,32,34]. To evaluate the role of CB1 and CB2 receptors in the JZL195 effect upon NTG-induced trigeminal hyperalgesia, NTG-injected rats received co-administration of JZL195 and a CB1 or a CB2 receptor antagonist 2 h after NTG.

To antagonize CB1 and CB2 receptors, we used AM251 ((N-(piperidin-1-yl)-5-(4-iodophenyl)-1-(2,4-dichlorophenyl)-4-methyl-1H-pyrazole-3-carboxamide)) and AM630 (6-iodo-2-methyl-1-[2-(4-morpholinyl)ethyl](4-methoxyphenyl)), respectively. Both antagonists were dissolved in the same JZL15 vehicle (2 mL/kg) and were administered at a dose of 1 mg/kg, i.p [35].

Four hours after NTG or vehicle administration, two behavioral test (details in Section 2.3 and Section 2.4) were performed consecutively (Figure 1) to evaluate: (i) locomotor and exploratory activity, (ii) anxiety-like behavior, (iii) nociception, and (iv) trigeminal hyperalgesia. The interval of 4 h between NTG administration and the behavioral tests was chosen since 4 h is the expected time of maximal expression of NTG-induced neuronal activation [30]. Rats were sacrificed at the end of the orofacial formalin test (Figure 1) to evaluate gene expression and CGRP serum levels (details in Section 2.5 and Section 2.6).

### 2.3. Open Field Test

The open field test was used to analyze the effect of JZL195 treatment on the locomotor activity, anxiety, and different types of behaviors, such as rearing and grooming. The open-field arena consisted of a 92 × 92 cm box (Ugo Basile), with grey opaque walls fit solidly into a grey non reflective base plate. Each rat was acclimatized to the test room for 60 min before testing. One minute before testing, each rat was placed into a cylinder chamber in the center of the arena for 1 min; this was aimed at reducing the locomotion resulting from experimenter handling [36]. After removing the cylinder, each rat was allowed to freely explore the arena and recorded for 10 min through a video camera placed over the arena. The test arena was cleaned prior to use and before each test with 70% ethanol. 

Measurement of the distance travelled in the entire arena, the time spent in the center, and rearing behavior were used to assess locomotor activity, anxiety, and exploration, respectively [37]. Additionally, we also evaluated the spontaneous grooming behavior, as an indicator of increased nociception [37]. The evaluation of these parameters was performed by a trained observer who was unaware of the treatment condition, using the ANY-Maze software (Ugo Basile, application version 4.99 g Beta). By means of the ANY-Maze software, the open-field arena was divided into 16 square units, identifying 4 squares as the center and 12 squares along the outer perimeter as the periphery.

### 2.4. Orofacial Formalin Test

Rats were acclimatized to the test chamber in the days before the orofacial formalin test for 10 min. The observation box was a 30 × 30 × 30-cm glass chamber with mirrored sides. A camera, recording face rubbing time for off-line analysis, was located at a distance of 50 cm from the box to provide a clear view of each rat.

Once the open field test was performed, each rat was moved to the orofacial formalin test room and 5 min later the animal underwent the subcutaneous injection of formalin (1.5%, 50 μL), an aqueous solution of 37% formaldehyde, performed into the right upper lip. Immediately after formalin injection, each rat was positioned into the observation box and its nocifensive behavior recorded for a 45-min period [30,38]. Face rubbing was measured by counting the seconds the animal spent grooming the injected area with the ipsilateral forepaw or hind paw 0–3 min (phase I, reflecting acute pain) or 12–45 min (phase II) after formalin injection. Phase II reflects the combined effects of afferent input and central sensitization. The observation time was divided into 15 blocks of 3 min each. Researchers who performed the evaluations were blind to treatments.

### 2.5. Tissue and Blood Sample Collection

Rats were sacrificed by decapitation after exposure to carbon dioxide [39] at the end of the formalin test and, immediately after, the blood samples were collected in a clot activator with gel separator serum tubes and centrifugated for 15 min at 1000× *g* at 2–8 °C. Serum CGRP levels were measured using a commercial enzyme-linked immuno-sorbent assay (ELISA) kit (Elabsciences, Cat.No: E-EL-R0135). The cervical spinal cord (CSC) and trigeminal ganglion (TG) ipsilateral to the formalin injection were quickly dissected out, rinsed in cold sterile 0.9% NaCl, placed in cryogenic tubes, and immediately frozen in liquid nitrogen. They were subsequently kept at −80 °C until rt-PCR processing.

### 2.6. rt-PCR

All procedures were performed under RNase-free conditions. Total RNA was extracted from samples with TRIzol^®^ (Invitrogen, Waltham, MA, USA), in combination with tissue homogenization by means of ceramic beads (PRECELLYS, Bertin Pharma). RNA quality was assessed using a nanodrop spectrophotometer (Euroclone) showing that the absorbance ratios (260/280 nm) ranged from 1.9 to 2.0 in all samples, indicating no significant protein (including of blood origin) contamination. cDNA was generated using the iScript cDNA Synthesis kit (BIO-RAD) following the supplier’s instructions. Gene expression was analyzed using the Fast Eva Green supermix (BIO-RAD). We evaluated the gene expression levels of CGRP, interleukin-6 (IL-6), and tumor necrosis factor-alpha (TNF-alpha) in CSC and TG ipsilateral to formalin injection. mRNA levels were measured by rt-PCR [30]. Primer sequences, obtained from the AutoPrime software (http://www.autoprime.de/AutoPrimeWeb; accessed on 1 September 2019), are reported in Table 1. Glyceraldehyde 3-phosphate dehydrogenase (GAPDH), whose expression remained constant in all experimental groups, was used for normalization. The amplification was performed through two-step cycling (95–60 °C) for 45 cycles with a light Cycler 480 Instrument RT-PCR Detection System (Roche) following the supplier’s instructions. All samples were assayed in triplicate and the ΔΔCt method was used to investigate the differences in gene expression levels.

### 2.7. Statistical Analysis

An a priori power analysis was conducted to determine the minimal sample size needed to obtain a statistical power of 0.80 at an alpha level of 0.05 (GPower 3.1). We hypothesized a difference in the total nociceptive response in the second phase of the orofacial formalin test (face rubbing time) between rats injected with NTG and rats injected with NTG vehicle of at least 27 s (169 ± 13.5; CT = 142 ± 18) and thus, we estimated a sample size of 6 rats in each experimental group with an effect size of 1.69. However, since the orofacial formalin test shows an intergroup variability, we used a maximum of 7 rats. Differences among groups were compared by ANOVA followed by the Tukey HSD post hoc test. Statistical significance was set at *p* < 0.05. All statistical analyses were performed using GraphPad Prism software (version 8). Data were expressed as mean ± standard deviation (SD). 

## 3. Results

### 3.1. Open Field Test

**Figure 2:**
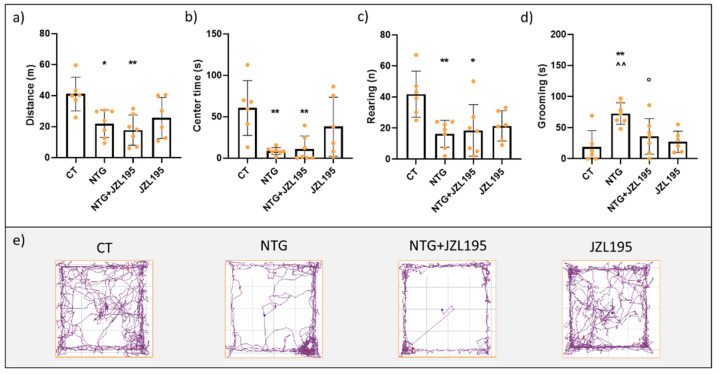
Open field test analysis. (**a**) Distance (expressed in meters) travelled in the apparatus; (**b**) time spent (expressed in seconds) in the center of the apparatus; (**c**) number of rearing; (**d**) time spent in grooming behavior (expressed in seconds); (**e**) representative track plots of the different experimental groups. Male Sprague–Dawley rats were treated with JZL195 at a dose of 3 mg/kg, dissolved in a volume of 2 mL/kg of vehicle (Tween-80/polyethylene glycol 200/saline), 2 h after NTG or vehicle injection. The locomotor activity, anxiety, and different types of behaviors were evaluated 4 h after NTG or vehicle injection. Data are expressed as mean ± SD. One-way ANOVA followed by Tukey’s multiple comparisons test; * *p* < 0.05 and ** *p* < 0.01 vs. CT; ^^ *p* < 0.05 vs. JZL195; ° *p* < 0.05 vs. NTG. CT: control group (*n* = 6); NTG: nitroglycerin group (*n* = 7); NTG + JZL195: nitroglycerin + JZL195 group (*n* = 7); JZL195: nitroglycerin vehicle + JZL195 group (*n* = 6).

Systemic administration of NTG reduced the locomotor activity, expressed as distance travelled compared with the CT group (Figure 2a). Additionally, it increased the anxiety-like behavior as indicated by a reduced time spent in the center of the open field (Figure 2b) and reduced the exploratory behavior, expressed as the number of rearing (Figure 2c), compared with the CT group. In the group treated with JZL195 and NTG vehicle (JZL195 group), we observed a pattern toward a decrease in these activities, which, however, did not reach statistical significance compared with the CT group (Figure 2a–c). When JZL195 was administered with NTG (NTG + JZL195 group), no significant changes were observed in the locomotor activity, time spent in the center of the open field and exploratory behavior. 

As regards the grooming behavior, indicative of increased nociception [37], NTG significantly increased the time spent in grooming compared to CT and this effect was significantly reduced by JZL195 administration (NTG + JZL195 group) (Figure 2d). 

**Figure 3:**
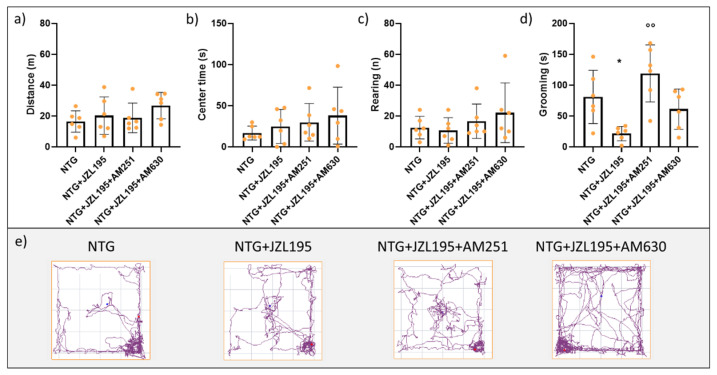
Open field test analysis. (**a**) Distance (expressed in meters) travelled in the apparatus; (**b**) time spent (expressed in seconds) in the center of the apparatus; (**c**) number of rearing; (**d**) time spent in grooming behavior (expressed in seconds); (**e**) representative track plots of the different experimental groups. Male Sprague–Dawley rats were treated with JZL195 (3 mg/kg) with or without the CBs antagonist (1 mg/kg) dissolved in a volume of 2 mL/kg of vehicle (Tween-80/polyethylene glycol 200/saline) 2 h after NTG injection. The locomotor activity, anxiety, and different types of behaviors were evaluated 4 h after NTG injection. Data are expressed as mean ± SD. One-way ANOVA followed by Tukey’s multiple comparisons test; * *p* < 0.05 vs. NTG; °° *p* < 0.01 vs. NTG + JZL195. NTG: nitroglycerin group (*n* = 6); NTG + JZL195: nitroglycerin + JZL195 group (*n* = 6); NTG + JZL195 + AM251: nitroglycerin + JZL195 + CB1 antagonist group (*n* = 6); NTG + JZL195 + AM630: nitroglycerin + JZL195 + CB2 antagonist group (*n* = 6).

To investigate the involvement of CB1 and CB2 receptors in the JZL195-induced effect, in a different experimental set, we co-injected AM251 or AM630 with JZL195 in NTG-challenged rats. In such condition, co-treatment of JZL195 with AM251 increased grooming behavior compared to the NTG + JZL195 group, while AM630 only induced a non-significant increase (Figure 3d). As regards the other evaluated parameters, no changes among groups were reported (Figure 3a–c). 

This result suggests that dual inhibition of FAAH and MAGL acts mainly at the nociceptive components, probably through the CB1 receptors. 

### 3.2. Orofacial Formalin Test

**Figure 4:**
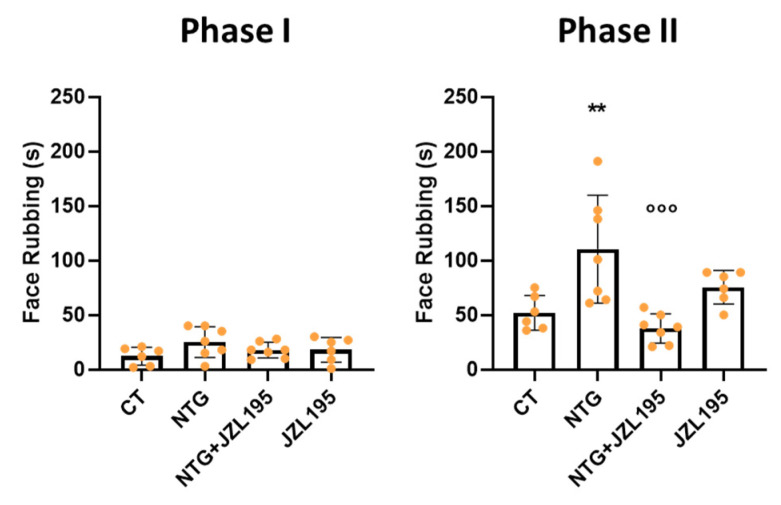
Phases I and II of the orofacial formalin test. Male Sprague–Dawley rats were treated with JZL195 at a dose of 3 mg/kg (i.p.) dissolved in a volume of 2 mL/kg of vehicle (Tween-80/polyethylene glycol 200/saline) 2 h after NTG or vehicle injection. The nocifensive behavior was evaluated 4 h after NTG or vehicle injection. Data are expressed as mean of face rubbing time (in seconds) ± SD. One-way ANOVA followed by Tukey’s multiple comparisons test; ** *p* < 0.05 vs. CT; °°° *p* < 0.001 vs. NTG. CT: control group (*n* = 6); NTG: nitroglycerin group (*n* = 7); NTG + JZL195: nitroglycerin + JZL195 group (*n* = 7); JZL195: nitroglycerin vehicle + JZL195 group (*n* = 6).

**Figure 5:**
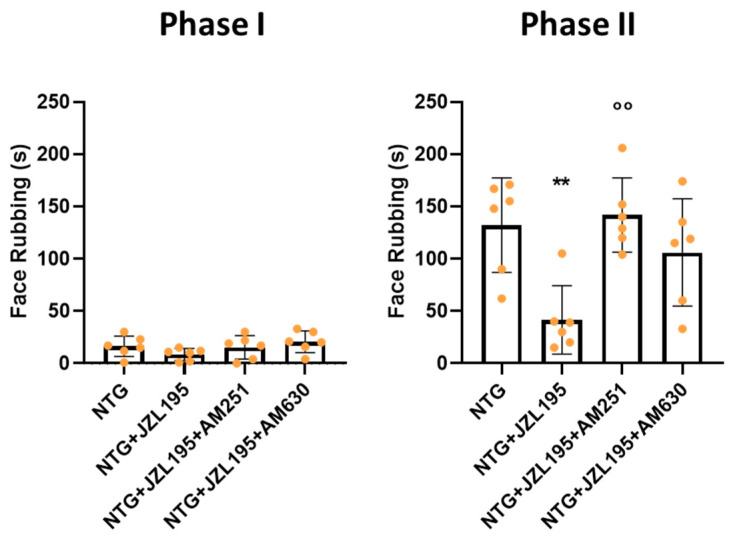
Phases I and II of the orofacial formalin test. Male Sprague–Dawley rats were treated with JZL195 (3 mg/kg) with or without the CBs antagonist (1 mg/kg) dissolved in a volume of 2 mL/kg of vehicle (Tween-80/polyethylene glycol 200/saline) 2 h after NTG injection. The locomotor activity, anxiety and different types of behaviors were evaluated 4 h after NTG injection. Data are expressed as mean of face rubbing time (in seconds) ± SD. One-way ANOVA followed by Tukey’s multiple comparisons test; ** *p* < 0.01 vs. NTG; °° *p* < 0.01 vs. NTG + JZL195. NTG: nitroglycerin group (*n* = 6); NTG + JZL195: nitroglycerin + JZL195 group (*n* = 6); NTG + JZL195 + AM251: nitroglycerin + JZL195 + CB1 antagonist group (*n* = 6); NTG + JZL195 + AM630: nitroglycerin + JZL195 + CB2 antagonist group (*n* = 6).

Orofacial formalin injection in NTG-treated rats provoked a significant increase in nocifensive behavior (face rubbing) compared with vehicle-treated controls (CT group) in the second phase of the test (12–45 min following formalin administration). JZL195 reduced formalin-induced nocifensive behavior (NTG + JZL195 group). No significant effect was observed when JZL195 was given in association with NTG vehicle (Figure 4). Co-administration of the CB1 inverse agonist, AM251, counteracted the anti-hyperalgesic effect of JZL195 in NTG-sensitized rats during phase II of the test (Figure 5). Similarly, an increase of face rubbing was seen when the inhibitor was co-administered with the CB2 antagonist, AM630, although not significantly. No significant differences between groups were noted during phase I of the test (Figure 5).

Here, the effects observed in the open field test of the dual FAAH/MAGL inhibitor on NTG-induced pain behavior are further confirmed. Moreover, JZL195 showed its ability in reducing NTG-induced trigeminal hyperalgesia, a hallmark of this animal model of migraine, through CB1 receptors.

### 3.3. Molecular and Biochemical Changes Induced by JZL195

**Figure 6:**
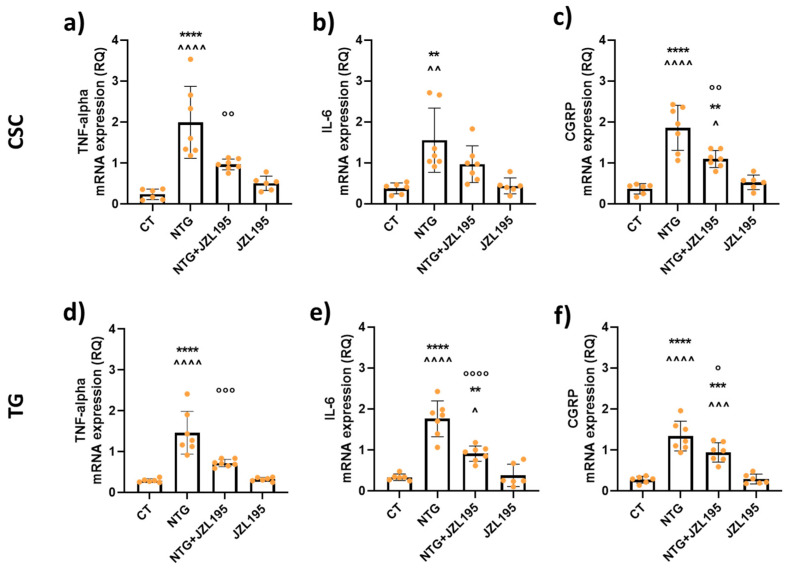
Cytokine gene expression levels in cervical spinal cord (CSC) and trigeminal ganglion (TG) ipsilateral to formalin injection. Male Sprague–Dawley rats were treated with JZL195 at a dose of 3 mg/kg (i.p.) dissolved in a volume of 2 mL/kg of vehicle (Tween-80/polyethylene glycol 200/saline). Rats underwent the orofacial formalin test 4 h after NTG and vehicle injection. All animals were sacrificed at the end of the formalin test to evaluate mRNA expression levels in peripheral and central areas. TNF-alpha (**a**,**d**), IL-6 (**b**,**e**), and CGRP (**c**,**f**) mRNA levels are expressed as relative quantification (RQ). Data are expressed as mean ± SD. One-way ANOVA followed by Tukey’s multiple comparisons test; ** *p* < 0.01, *** *p* < 0.001, and **** *p* < 0.0001 vs. CT; ^ *p* < 0.05, ^^ *p* < 0.01, ^^^ *p* < 0.001, and ^^^^ *p* < 0.0001 vs. JZL195; ° *p* < 0.05, °° *p* < 0.01, °°° *p* < 0.001, and °°°° *p* < 0.0001 vs. NTG. CT: control group (*n* = 6); NTG: nitroglycerin group (*n* = 7); NTG + JZL195: nitroglycerin + JZL195 group (*n* = 7); JZL195: nitroglycerin vehicle + JZL195 group (*n* = 6).

TNF-alpha, IL-6, and CGRP gene expression significantly increased in CSC and TG ipsilateral to formalin injection in rats treated with NTG (Figure 6), suggestive of increased pain and inflammation. JZL195 treatment prevented the increase of CGRP, IL-6 and TNF-alpha mRNA levels in both areas compared to the NTG group (Figure 6). No effect on gene transcription was observed when JZL195 was given with NTG vehicle. 

**Figure 7:**
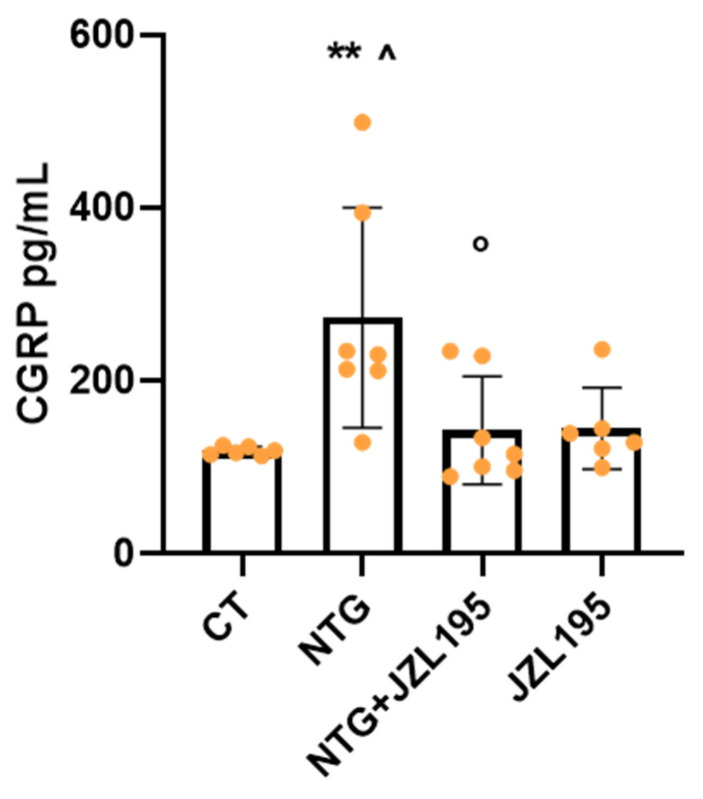
CGRP serum levels (pg/mL). Male Sprague–Dawley rats were treated with JZL195 at a dose of 3 mg/kg (i.p.) dissolved in a volume of 2 mL/kg of vehicle (Tween-80/polyethylene glycol 200/saline). Rats underwent the orofacial formalin test 4 h after NTG and vehicle injection. All animals were sacrificed at the end of the formalin test and blood collected evaluate CGRP serum levels. Data are expressed as mean ± SD. One-way ANOVA followed by Tukey’s multiple comparisons test; ** *p* < 0.01 vs. CT; ^ *p* < 0.05 vs. JZL195; ° *p* < 0.05 vs. NTG. CT: control group (*n* = 6); NTG: nitroglycerin group (*n* = 7); NTG + JZL195: nitroglycerin + JZL195 group (*n* = 7); JZL195: nitroglycerin vehicle + JZL195 group (*n* = 6).

Finally, NTG induced a significant increase in CGRP serum levels, which was significantly decreased after JZL195 treatment (Figure 7). 

These data indicate that JZL195 is able to antagonize the NTG-induced effects related to pain perception as reported above in both behavioral tests.

## 4. Discussion

The endocannabinoid system may control the release of several mediators involved in migraine pain (see [24] for a review), including CGRP and pro-inflammatory cytokines. Within the trigeminal system, cannabinoid CB1 receptor immunoreactive neurons are mainly found in the trigeminal nerve [40]. It was demonstrated that trigeminal CB1 receptors’ activation can inhibit CGRP release [41] as well as induce dural vasodilatation [42]. We previously showed in rats that pre-treatment with AEA (20 mg/kg, i.p.) significantly reduced neuronal activation in the trigeminal nucleus caudalis and decreased NTG-induced hyperalgesia in the plantar formalin test [43]. The analgesic effects of AEA were reported in the same animal model also by another group [19]. Similarly, treatment with a specific CB2 agonist was efficacious in reducing NTG-induced hyperalgesia [44]. Kilinc et al. [45] confirmed, by using the same animal model, that the elective ligands targeting CB1 and CB2 receptors may provide novel and effective treatment strategies against migraine. Moreover, we showed that NTG-induced hyperalgesia is associated with increased activity of the hydrolyzing enzymes MAGL and FAAH and increased density of CB binding sites in the mesencephalon and an increase of FAAH activity in the hypothalamus associated with augmented CB protein expression [46]. These findings suggest a dysfunction of the endocannabinoid system in migraine pathophysiology and that pharmacological modulation of this system can be useful for the treatment of migraine pain. The potentiality of increasing endocannabinoid system activity through inhibition of FAAH or MAGL has been tested in animal models of migraine [24]. In previous studies, we showed that systemic administration of URB937 (a peripherally restricted FAAH inhibitor) and URB597 (a global FAAH inhibitor) reversed trigeminal hyperalgesia elicited by NTG administration in rats [27,29,30]. We further showed that the anti-hyperalgesic effects of URB937 are CB1 mediated, thus confirming the role of the peripheral CB1 receptor in the anti-hyperlgesic effect [6,39]. Similarly, the MAGL inhibitors URB602 and JZL184 reduced NTG-induced hyperalgesia either of spinal or trigeminal origin [28]. By contrast, both MAGL inhibitors increased formalin-induced hyperalgesia in the trigeminal area in rats not subjected to NTG challenge, suggesting a diverse effect of 2-AG in trigeminal/extra-trigeminal in the presence/absence of the NTG challenge [28]. In the present study, we did not observe an anti-hyperalgesic effect when JZL195 was administered in animals not subjected to the NTG challenge, probably because of its dual action which presumably resulted in balanced activity of AEA and 2-AG. By contrast, JZL195 exerted anti-hyperalgesic effects in the orofacial formalin test when administered to NTG-challenged rats. However, it must be noted that JZL195, beside AEA and 2-AG, may also influence the levels of other endocannabinoid congeners. Specifically, a pronounced elevation of OEA and PEA brain levels was reported, due to JZL195’s inhibitory activity at FAAH [47]. Here, we further show that the anti-hyperalgesic effects of JZL195, probably related to endocannabinoid system modulation, require the CB1 receptor, thus confirming the role of this receptor subtype in the analgesic response. However, a role of CB2 receptors, as well as other receptors, should not be excluded. Additional studies must be performed in this direction.

The effects of this dual inhibitor to revert pain sensitization were observed also in other pain models. For instance, JZL195 was shown to produce a dose-dependent reversal of the chronic constriction injury (CCI)-induced reduction in mechanical paw withdrawal threshold, 2 h post injection [13]. It decreased mechanical allodynia and thermal hyperalgesia in an animal model of inflammatory pain [9], 1 and 2 h post injection. 

Interestingly, in parallel with its inhibitory effect on NTG-induced hyperalgesia, JZL195 also reduced CGRP serum levels and CGRP gene expression in specific peripheral and central areas relevant for migraine pain. The findings are in agreement with previous data showing that a chiral analog of AEA significantly reduced the increase in CGRP levels induced by NTG in rats’ plasma, TG neurons, and brainstem [45]. It is worth noting that in an isolated skin preparation [48], AEA inhibited CGRP release at low concentrations, but it increased the neuropeptide’s levels at higher concentrations. Similarly, intraplantar administration of 2-AG blocked the second phase of formalin-evoked pain behavior in rats, by a CB2-mediated mechanism [5]. The exact mechanism underlying JZL195’s anti-nociceptive activity in our animal model is not known and the present data do not fully contribute to unveil this aspect. However, it can be speculated that inflammation in the dura and TG could stimulate the localized release of endocannabinoids and PEA from primary sensory afferents as well as from non-neural cells, including macrophages and mast cells. Of note, MAGL and FAAH were found in microglia and astrocytes [49]. 

In addition, our results suggest a modulatory effect of the endocannabinoid pathways, by sustaining the tone of endocannabinoid and congener lipids [47], on CGRP, whose release from both central and peripheral trigeminal terminals increases the activity of second-order neurons of the trigeminal nucleus caudalis, causing trigeminal hyperalgesia. Since AEA and 2-AG are known to act via a range of partially overlapping targets [50,51], JZL195’s effects are probably mediated by both CB1 and CB2 receptors [9] as partially displayed by our data. 

Alternatively, the expected increased availability of AEA after JZL195 might induce desensitization of the transient receptor potential vanilloid 1 channel [52], probably via the inhibition of the release of nitric oxide (NO) [53] or of neuropeptides from primary afferents at the spinal level [48]. Further, the potential effect of 2-AG or PEA on the PPARs receptors at the meningeal level could contribute to a reduction of NTG-induced inflammation [54,55].

Accordingly, in the present study, we reported that the decrease in CGRP plasma levels and gene expression was associated with a significant reduction of TNF-alpha and IL-6 gene expression in peripheral and central areas involved in migraine pain. Indeed, endocannabinoids have been shown to downregulate inflammation in numerous experimental models, such as inflammatory pain [5,56]. In agreement with our findings, the MAGL inhibitor JZL184 counteracted LPS-induced increases in IL-1β, IL-6, and TNF-alpha in the rat frontal cortex [57]. Likewise, in an animal model of multiple sclerosis, increasing endocannabinoid tone with UCM-707—an AEA uptake inhibitor—resulted in a significant reduction of IL-1β levels and an increase in IL-10 levels [5,56,58]. However, further experimental data are needed to confirm this speculation and to dissect the potential mechanisms.

**Figure 8:**
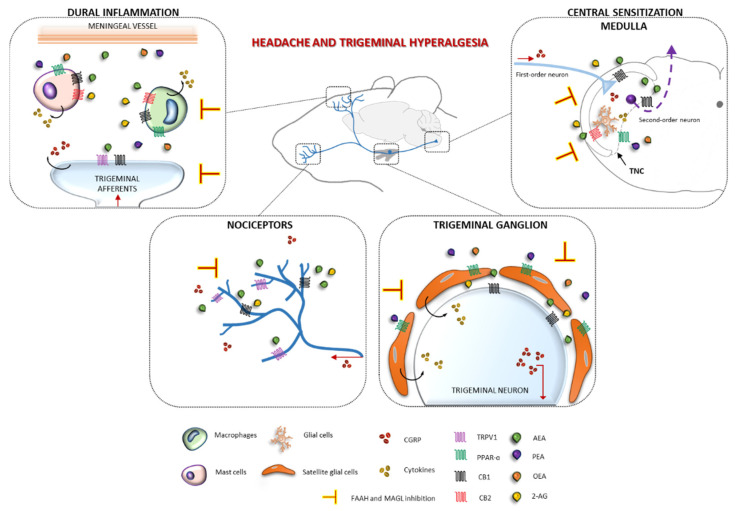
Trigeminal afferents, arising in the trigeminal ganglion, convey sensory information from the intracranial and extracranial sites to the trigeminal nucleus caudalis (TNC), causing trigeminal hyperalgesia. Inflammation induced by NTG in the dura and trigeminal ganglion stimulates the local release of endocannabinoids and congeners from primary sensory afferents and non-neural cells, including macrophages and mast cells. Endocannabinoids and congeners (PEA, OEA), released from membrane’s lipids, may be initiated by calcium entry in nociceptors and macrophages. Successively, lipids may activate CB1 and/or CB2 or PPAR-α, modulating membrane excitability and calcium signals in primary afferent terminals, inhibiting macrophage activation and mast-cell degranulation and thus inflammation. Alternatively, AEA might induce desensitization of the transient receptor potential vanilloid 1 channel (TRPV1), probably via inhibition of the release of nitric oxide or of neuropeptides from primary afferents at the spinal level.

The putative biological effects of JZL195 in migraine pain and the areas of possible crosstalk with CGRP and inflammatory mediators are schematically illustrated in Figure 8.

Beside the orofacial formalin test, used to measure NTG-induced trigeminal hyperalgesia, we adopted the open field test as a tool to evaluate locomotor, exploratory, and anxiety-like behavior in the NTG-based migraine model. Here, we found an alteration of these parameters in the NTG-treated rats, which is in line with previous studies showing the involvement of NO in the modulation of anxiety [59,60] and locomotor activity [61]. Contrary to our results, Sufka and collaborators [62] reported increased locomotor activity 2 h after a single injection of NTG in rats compared to control animals. The discrepancy with our data is probably related to the different time points (2 h vs. 4 h post NTG injection in our paradigm) and the different apparatus and methodology applied (light–dark box used by Sufka et al. vs. open field used in our study). By means of the open field test, we also reported that NTG-challenged rats spent more time in spontaneous grooming behavior, indicative of increased nociception [37]; this behavior was reduced when the animals that underwent JZL195 treatment. Together with the findings obtained in the formalin test, we showed that JZL195 effectively exerts anti-hyperalgesic effects by acting on pain and inflammatory pathways, confirming data observed in other pain models [10,31]. The FAAH/MAGL inhibitor at the 3 mg/kg dose did not influence the locomotion or other anxiety-like behavior changes induced by NTG, nor did it affect these activities when used alone (NTG vehicle), confirming previous data [13]. By contrast, higher doses of JZL195 (10 mg, 20 mg/kg, i.p.) induce side effects in rodents, such as impaired motor coordination in the rotarod test, catalepsy in the bar test, and reduced locomotion (sedation) [13,63], probably mediated by elevation in the levels of AEA and 2-AG [31].

Taken together, our data suggest that JZL195 may induce effects that are relevant for migraine pain, and it might cause different biological effects (i.e., increase of AEA and 2-AG levels) by specific dose-dependent changes in central and peripheral areas [32]. We suppose that JZL195 could induce effects related to a selective increase of AEA and/or 2-AG or congeners in some definite regions, modulating nociception, rather than others. For instance, previous studies have shown that the administration of the selective MAGL inhibitor JZL184 reduces anxiety-like behaviors in adult rodents [64,65,66], but it may selectively elevate cerebral 2-AG in vivo and reduce locomotor activity in rats [34]. We observed a lack of an effect of JZL195 on anxiety-like and motor behaviors at a dose of 3 mg/kg, while JZL195 at higher doses (15 and 30 mg/kg, i.p.) decreases motor activity and rearing numbers compared with JZL184 [34], suggesting that inhibition of the 2-AG catabolic enzyme by JZL195 may exert actions that differ from those induced by the selective JZL184. Thus, the available studies suggest that the effects of JZL195 are very complex, and its action may depend on the testing environment, the time of testing, dosages, and endocannabinoids levels in specific brain regions. 

### Limitations of the Study

Here, we demonstrated that the use of FAAH/MAGL inhibition may be a potential therapy for migraine pain, in particular, the modulation of spontaneous grooming behavior suggests a direct effect on regions implicated in trigeminal nociception. However, additional research is needed to investigate the changes in endocannabinoid and congener levels and the trigeminal level and evaluate if their distribution is restricted, for instance, to one or more peripheral regions (e.g., meninges, trigeminal ganglion) or central areas (e.g., trigeminal nucleus caudalis), to consider an acceptable benefit-to-risk ratio.

The findings provide further evidence in favor of a possible role of the endocannabinoid system in migraine pathophysiology but do not provide conclusive data regarding the specific importance of dual FAAH/MAGL inhibition as a potential migraine treatment versus single enzymatic inhibition. 

## 5. Conclusions

The present study confirms that the modulation of the catabolic pathways of AEA and 2-AG reduces pain signaling in animal models of migraine and it contributes some of the molecular mediators implicated in this activity. Both endocannabinoids and congeners can independently regulate pain sensation [6,67] via different and still undefined mechanisms, and the individual inhibition of their catabolic pathway has already proved beneficial in animal models specific for migraine [26,30]. Whether the simultaneous inhibition of AEA and 2-AG catabolic pathways using dual inhibitors may exert a synergistic effect in the migraine mechanism is still to be demonstrated, but the present findings contribute to further reinforce the role of the endocannabinoid system in migraine and provide yet another modulatory modality. 

## Figures and Tables

**Table 1:** This is a table. Tables should be placed in the main text near to the first time they are cited.

Gene	Forward Primer	Reverse Primer
GAPDH	AACCTGCCAAGTATGATGAC	GGAGTTGCTGTTGAAGTCA
CGRP	CAGTCTCAGCTCCAAGTCATC	TTCCAAGGTTGACCTCAAAG
IL-6	TTCTCTCCGCAAGAGACTTC	GGTCTGTTGTGGGTGGTATC
TNF-alpha	CCTCACACTCAGATCATCTTCTC	CGCTTGGTGGTTTGCTAC

## Data Availability

The data presented in this study will be made available upon reasonable request.
